# Controlling Social Stress in Virtual Reality Environments

**DOI:** 10.1371/journal.pone.0092804

**Published:** 2014-03-26

**Authors:** Dwi Hartanto, Isabel L. Kampmann, Nexhmedin Morina, Paul G. M. Emmelkamp, Mark A. Neerincx, Willem-Paul Brinkman

**Affiliations:** 1 Department of Intelligent Systems, Delft University of Technology, Delft, The Netherlands; 2 Department of Clinical Psychology, University of Amsterdam, Amsterdam, The Netherlands; 3 Department of Psychology, King Abdulaziz University, Jeddah, Saudi Arabia; 4 TNO Human Factors, Soesterberg, The Netherlands; ICREA-University of Barcelona, Spain

## Abstract

Virtual reality exposure therapy has been proposed as a viable alternative in the treatment of anxiety disorders, including social anxiety disorder. Therapists could benefit from extensive control of anxiety eliciting stimuli during virtual exposure. Two stimuli controls are studied in this study: the social dialogue situation, and the dialogue feedback responses (negative or positive) between a human and a virtual character. In the first study, 16 participants were exposed in three virtual reality scenarios: a neutral virtual world, blind date scenario, and job interview scenario. Results showed a significant difference between the three virtual scenarios in the level of self-reported anxiety and heart rate. In the second study, 24 participants were exposed to a job interview scenario in a virtual environment where the ratio between negative and positive dialogue feedback responses of a virtual character was systematically varied *on-the-fly*. Results yielded that within a dialogue the more positive dialogue feedback resulted in less self-reported anxiety, lower heart rate, and longer answers, while more negative dialogue feedback of the virtual character resulted in the opposite. The correlations between on the one hand the dialogue stressor ratio and on the other hand the means of SUD score, heart rate and audio length in the eight dialogue conditions showed a strong relationship: *r*(6) = 0.91, *p* = 0.002; *r*(6) = 0.76, *p* = 0.028 and *r*(6) = −0.94, *p* = 0.001 respectively. Furthermore, more anticipatory anxiety reported before exposure was found to coincide with more self-reported anxiety, and shorter answers during the virtual exposure. These results demonstrate that social dialogues in a virtual environment can be effectively manipulated for therapeutic purposes.

## Introduction

Social anxiety disorder, also commonly referred as social phobia, is one of the most prevalent mental disorders [1]. People with social phobia experience a strong fear of being judged negatively by others and of being embarrassed in social situations (e.g., talking to other people or eating or drinking in front of other people). The gold standard to treat patients with social phobia is cognitive behaviour therapy with the central component being gradual exposure *in vivo*, whereby patients are gradually exposed to anxiety provoking real-life situations until they habituate to the anxiety provoking social situations. Although exposure *in vivo* is an effective treatment for most patients, it is also associated with some limitations, such as the limited therapeutic control over different aspects of exposure and a relatively high number of drop outs as some patients are not willing to get exposed to feared situations [2]–[4].

Virtual Reality Exposure Therapy (VRET) has been proposed as an effective alternative to overcome these shortcomings of exposure in vivo [5]–[9]. Exposure in virtual reality (VR) makes the control of exposure elements more manageable since the patient is exposed in a controlled Virtual Environment (VE) where the parameters of anxiety evoking stimuli can be changed and manipulated by the therapist. Current VRET systems used for social phobia patients mainly focus on recreating a social scene setting, such as public speaking scenarios, clothing shops, public transport, or restaurants. At the start of the treatment, an anxiety hierarchy of anxiety-arousing social situations is established. This hierarchy is then used to order the VR situations the patient will be gradually exposed to, starting with less anxiety-arousing situations and eventually moving to more anxiety-arousing situations as treatment progresses.

Even though several studies [3], [4], [10], [11] have reported promising initial efficacy findings for VRET for social phobia, the used VR systems mainly allow the therapist to control social anxiety only by moving between different VR situations. Hence during the actual exposure in the VR situation, the therapist has limited ability to introduce or remove anxiety-evoking stimuli in a VR world. Based on research on other anxiety disorders, this ability might prove useful for the treatment of social phobia as well [12]. For example, in VRET for fear of flying, a therapist is given the opportunity to make relevant changes of the virtual world in use if appropriate, such as switching on the sign *fasten your seatbelts*, flying under different weather conditions, or letting the pilot make a specific announcement [13]. In VRET for fear of heights, the therapist can move patients to a higher step of a virtual staircase, or place them closer to the edge of a balcony [14]. With regard to VRET for social phobia, however, little attention has been given to this aspect of the treatment. We argue that manipulating the dialogue between the patient and a virtual character can increase the efficacy of VRET for social phobia. For example, by having the virtual character responding on the behaviour of the patients in the dialogue, the therapist can directly address the fear of being evaluated negatively by others.

Affective feedback plays a key role in dialogues between humans, and can elicit for example defensive or supportive listener’s feedback responses [15]. Furthermore, the listener can in turn actively influence the emotional state of the speaker, as is fundamental to empathic listening technique [16]. In the long term, human interaction influences individuals’ self-esteem as it feeds into their reflected appraisal process [17], i.e. the way they imagine how other people see or judge them [18].

Furthermore, a conversation between a human and virtual characters, which mirrors a role-play between human and human dialogue conversation, can influence the emotional state of the human, as has been demonstrated in prior research with VR systems [19], [20]. Yet, the emotion manipulation and evaluation of the stressor stimuli until now has always covered the entire conversation and has not been directed at isolated sections within a conversation. Accordingly, the aim of this study was to investigate whether it is possible to induce anxiety in a virtual environment by manipulating the dialogue feedback responses between a human and virtual characters as this could benefit VRET.

### Related Work

For exposure in vivo, therapy manuals [21] often suggest scenarios that include social interaction, i.e. a dialogue with other individuals, for example, asking multiple people for directions to an obvious location, asking a person at a bar whether he has seen a specific movie and asking if he knows the main actors in the movie, renting a DVD and immediately asking your money back as you do not have a DVD player. When it comes to exposure in virtual reality, most studies focus mainly on public speaking scenarios [2], [11], [22]–[24]. However, in recent studies, other social scenarios have also been successfully developed, such as a restaurant scenario [25], interaction inside public transport [10], clothing store [26], train and bus station [25], [26], a bar scenario [10], [27], formal job interview [26], [28] and a blind date scenario [26]. These VRET systems allow therapists to use a variety of virtual social scenes to expose patients to different social situations thereby following the order set by the fear hierarchy. Some patients however might feel unable to conduct exposure, or opposite, do not experience enough anxiety or discomfort. In these cases therapist manuals [21] for exposure in *vivo* advise therapists to show an adequate degree of flexibility and modify the exposure accordingly, e.g. change the topic of the presentation, bring in new audience members, or interrupt the patient at various points in their presentation. The aim is to establish an optimal level of anxiety during the anticipation phase of exposure. For example, some manuals [21] have even suggested as a general rule to have an anxiety level of somewhere between 5 and 7 on an 11-point scale from 0 (no anxiety) to 10 (extreme anxiety). However, this level might be very much patient dependent.

For exposure in virtual reality, flexibility has been sought in changing the body posture of the members of a virtual audience [29], eye gaze of the virtual character [26], [28], distance between virtual character and the patient [30], [31], the facial expression [30], [32], attitude [29], [33] or personality [26], [34] of the virtual character. However, less research attention has been devoted to control the verbal element of the interaction, i.e. the content of the dialogue. Most VRET systems that recreate social situations provide no or only limited verbal responses of the virtual character, while there is clear support that humans respond in a similar manner to a computer that acts as social actor as they would do when interacting with other humans [19], [32], [35], [36]. Some have also studied this phenomenon in the context of dialogues with virtual humans. This has been done not only to study social anxiety [26], [29], [30], specifically public speaking anxiety, but also schizophrenia [31]. For example, Ku et al. [31] demonstrated that virtual humans could engage schizophrenic patients with limited dialogue only. Slater et al. [20] demonstrated that people were aroused when virtual humans communicated with them. They found that people with more social anxiety also experienced more stress compared to people with less social anxiety when engaged in an active conversation with virtual humans. Using semi-scripted conversations and speech recognition, ter Heijden and Brinkman [37] showed that it is possible to create 5 minute elaborate conversations between the patients and virtual humans in virtual reality as part of a question and answer session after a small presentation. The use of semi-scripted conversations has now also been used to recreate conversations in other social situations such as having a conversation with a stranger at a bus stop, buying a t-shirt, a bra or baby clothes in a shop and dining with a blind date [26].

Virtual humans have also been used for educational purposes. Interestingly, here the effect of feedback has been studied. For example, receiving positive instead of negative feedback from a digital assistant can speed up the students learning time [38], [39]. Also pedagogical agents that give positive or empathic feedback can enhance the student’s interest in a topic and their self-efficacy [40], [41]. As social situations in virtual reality with relative long dialogue interaction provide multiple opportunities to give patients positive or negative feedback, it would be an ideal phobic stressor for a VRET system. Another major advantage is that the situation can be changed while the patient is being exposed. This would give the therapist non-interruptive intervening possibilities to get the patient’s anxiety to an ideal level for exposure to work. Hence, this paper presents two studies that examine the following hypotheses underlying this idea:

- **H1**: Different social dialogue situations are able to elicit different levels of anxiety.
- **H2**: In a social dialogue situation, the ratio of positive and negative responses from a virtual human proportionally affects the human anxiety level whereby a dialogue with mainly negative responses elicits more anxiety than a dialogue with mainly positive responses.
- **H3**: After a dialogue with a virtual human that contained responses mainly of one affect polarity, anxiety will change correspondingly if the dialogue continued with fewer response of this affect polarity. In other words, the level of anxiety can be controlled *on-the-fly* by manipulating the dialogue polarity.
- **H4**: Individual’s degree of social anxiety is positively related to the amount of elicited anxiety when exposed to a dialogue stressor.

## Method (First study: Social Scene Experiment)

### Ethics Statement

The first study was approved by Delft University of Technology Human Research Ethics Committee. Prior to the experiment, written informed consent was obtained from all participants. Furthermore, for publication policy, the individual in this manuscript has also given written informed consent (as outlined in the PLOS consent form) to publish case details. After the experiment, participants received a chocolate bar and drink (tea, coffee or warm chocolate) as a token of appreciation for their participation.

### Experiment Design

**Figure 1:**
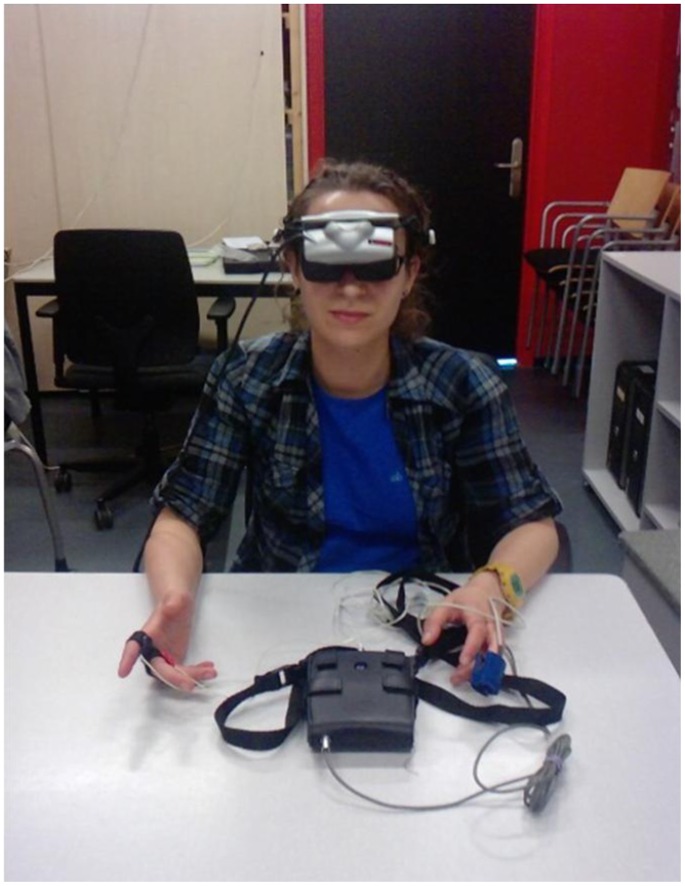
Setup of the experiment.

**Figure 2:**
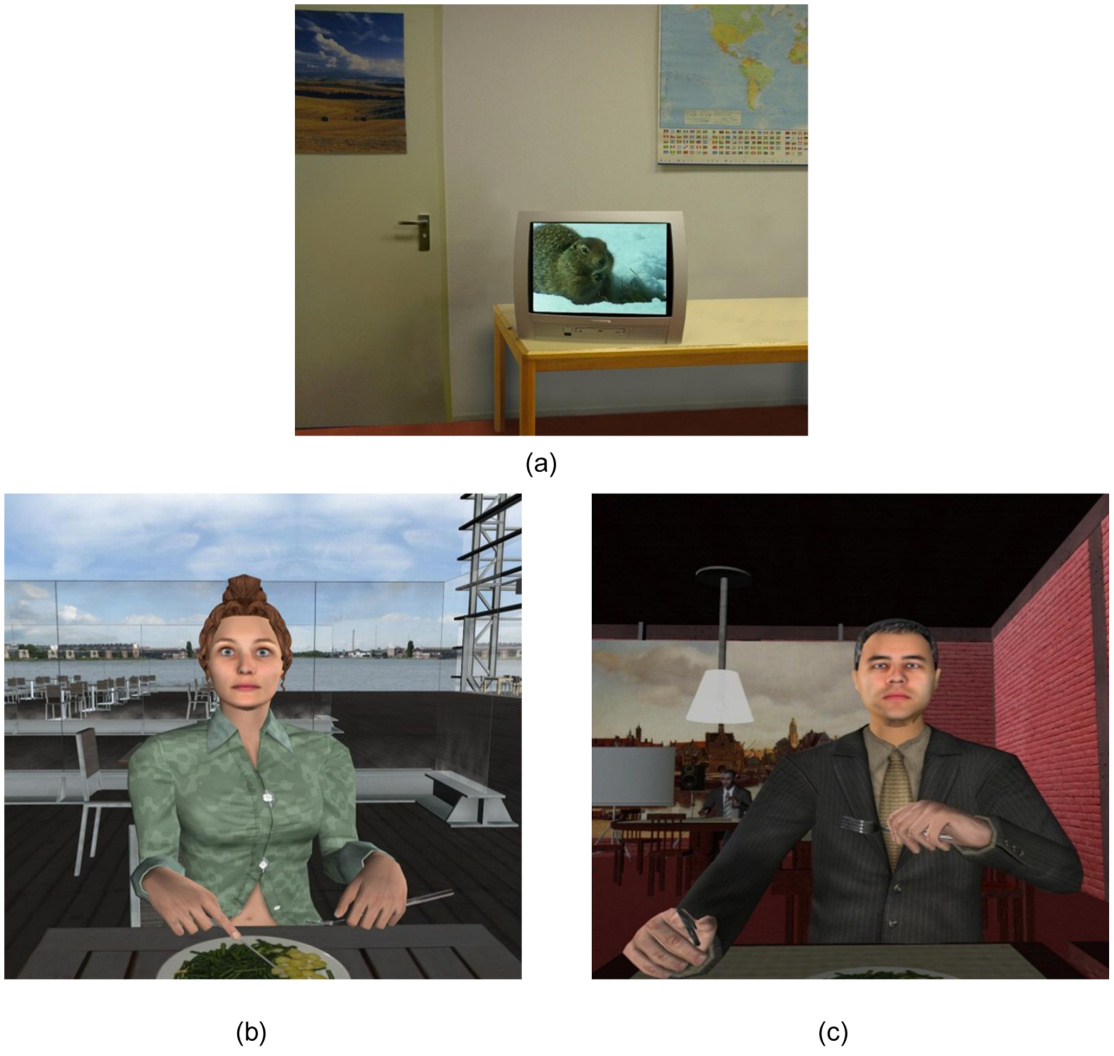
Virtual neutral world (a), virtual blind date (b) and virtual job interview (c).

In order to study the effect of various VR scenes on anxiety level, a within-subjects experiment was conducted. Figure 1 depicts the experimental setup. All participants were exposed to three different VR scenarios: a neutral virtual world [42] where participants were seated in front of a television showing a documentary about wildlife (Figure 2(a)), a blind date (Figure 2 (b)), and a job interview session with virtual humans (Figure 2 (c)).

### Participants

Sixteen participants (5 females and 11 males) were recruited in the first experiment that was approved by the ethics committee of Delft University of Technology. Fourteen participants were recruited from Delft University of Technology and two from Utrecht University. The age of the participants ranged from 19 to 27 years (*M* = 22.44, *SD* = 2.42). All participants reported to have seen 3D stereoscopic images or movies, yet none of them reported to have been exposed to virtual reality environments before. Furthermore, all participants were naïve with respect to the hypothesis.

### Measurements

#### SUD score

The levels of anxiety in the virtual environments were measured with the Subjective Units of Discomfort (SUD) developed by Wolpe [43]. The SUD scale measures levels of anxiety on a scale from zero (“no anxiety at all”) to 10 (“the highest level of anxiety that you can imagine”).

#### Presence

Sense of presence in the virtual reality environment was assessed with the Igroup Presence Questionnaire (IPQ) developed by Schubert et al. [44]. The IPQ is composed of 14 items rated on a seven-point Likert scale. The scores on the 14 IPQ items are mapped onto three subscales, namely *Involvement* (the awareness devoted to virtual environment), *Spatial Presence* (the relation between the virtual environment and the physical real world), and *Experienced Realism* (the sense of reality attributed to the virtual environment). IPQ also contains one item that assessed participants’ general feeling of being in the virtual environment.

#### Heart rate

It was expected that the heart rate would increase if people were feeling anxious in a daunting or frightening situation. To measure the elicited fear responses in this experiment, heart rate of the participants was monitored continuously. The heart rate was recorded with a Mobi8 device from TMSI with an Xpod Oximeter. The participant inserted a finger into an adult articulated finger sensor.

### Procedure and Apparatus

At the start of the experiment participants received a short introduction about the overall aim of the study and filled in a basic questionnaire about their age, education and signed a consent form. Participants were not informed about the different VR scenario conditions.

The main part of the experiment consisted of three different VR scenarios, one passive interaction with neutral VR world and two active free speech interactions with virtual humans (Figure 2 (b) and (c)). To control for possible learning effect, the order of the two active interaction scenarios (conditions) was counter balanced.

A baseline measurement was obtained by exposing participants to the neutral VR world. This also allowed them to become familiar with the VR exposure. This session lasted for two minutes and at the end of the session participants were asked to rate their SUD score. In the other conditions, participants had two sessions (five minutes each) active free speech interaction with a virtual human in VR. The two VR social scenes that were used in the experiment were: (a) meeting a blind date in a musical building’s restaurant (where the virtual blind date’s gender was adapted for participants’ gender) and (b) having a job interview for a restaurant server position. Job interview and blind date scenes were selected as relevant social scenes to elicit social anxiety since both social scenes are often suggested for in-session exposures in the cognitive-behavioural group therapy [45]. At the end of this session, participants were asked to rate their SUD score. After these two sessions, participants filled in the IPQ. The entire experiment took about 25 minutes.

All participants were exposed to a virtual reality environment using the Delft Remote Virtual Reality Exposure Therapy (DRVRET) system [26]. This system allows participants to engage in a free speech dialogue with virtual characters while being monitored by a therapist, in this case the therapist was replaced by the researcher. Both, the blind date and the job interview scenes contained between 35–50 pre-recorded sentences, which the researcher could use to let the virtual human respond to the participants’ answer or comment with the aim of engaging the participants in to a 4 to 5 minutes conversation. As a fallback strategy, each dialogue also has a number of dialogue independent responses, which could be used in case the participants’ answer or comment did not match one of the dialogue dependent responses. To avoid an ever widening dialogue tree, as each virtual character response can move the dialogue into new directions, the dialogues were written in such a way that they merged back into the dialogue’s main story line [37].

The software package Vizard v3.0 was used for the visualization of the virtual room and virtual human. Animations for virtual humans were created using 3D Studio Max using a keyframe method. The hardware used was a Dell Precision T3400 with Intel quad core Q6700 @ 2.66 Ghz, 4 GB of RAM, with NVidia Geforce Quadro FX 4600 graphic card running on Windows 7×64 bit as patient’s computer and a Toshiba Satellite L300 running on Windows 7×32 bit as therapist’s computer. Participants sat behind a table equipped with a microphone and wore the eMagin Z800 Head Mounted Display (HMD) on 800×600 pixels resolutions, with 40 degrees diagonal Field of View, and built-in 3DOF tracker tracked at a 125 Hz update rate and the sound was played through desk-mounted speakers.

## Results

A series of multivariate and univariate analyses were conducted. Where the sphericity assumption was violated a Greenhouse-Geisser correction was applied. To control for possible inflations of Type I Error, *post-hoc* analyses were conducted with Sidak correction.

To get a general understanding of the experienced presence level and to examine whether the virtual world in this experiment established a reasonable level of presence to evoke the anxiety, the overall IPQ score was compared to the online IPQ data set (downloaded on February 9^th^, 2013. For comparison data see: http://www.igroup.org/pq/ipq/data.php) for stereo HMD visual stimuli. The overall IPQ rating (*M* = 52.44, *SD* = 3.05) in this experiment was significantly higher (*t*(51) =  −3.22, *p* = 0.002) than the overall IPQ online data set (*M* = 38.16, *SD* = 17.53), which suggest that the participants were more immersed than the level reported in other virtual worlds.

In order to test the overall effect of different social scenes on levels of anxiety (H1), a repeated-measures MANOVA was conducted, with different social scenes (neutral VR world, virtual blind date and virtual job interview) as the independent within-subjects variables. The SUD score and heart rate were used as dependent variables. The results showed a significant effect of different virtual social scenes on anxiety levels, (*F*(4,12) = 16.94, *p*<0.001, *η^2^* = 0.85). Furthermore, univariate analyses found significant effects (*F*(2,30) = 36.65, *p*<0.001, *η^2^* = 0.71) on the SUD score and heart rate (*F*(2,30) = 23.52, *p*<0.001, *η^2^* = 0.61).

Next, *post-hoc* tests with Sidak correction were performed on the SUD score rating and heart rate in all three conditions. The results are presented in Table 1. Both, exposure to the blind date scene and the job interview scene were associated with significantly higher SUD levels. Furthermore, exposure to the job interview scene was associated with higher SUD levels than exposure to the blind date scene. A similar pattern was found in the heart rate measurement, participants’ heart rate in the job interview scene was higher than in the blind date scene and the participants’ heart rate in the blind date scene was again higher than in the neutral VR world.

**Table 1:** Comparison between different conditions on SUD score and heart rate.

Measurement		*M1*(*SD*)^a^	*M2*(*SD*)^b^	*t*	*df*	*p*
Condition 1	Condition 2					
**SUD score (0–10)**						
Neutral	Blind date	2.38 (0.89)	3.69 (1.01)	−5.55	15	<0.001
Neutral	Job interview	2.38 (0.89)	4.56 (1.03)	−7.89	15	<0.001
Blind date	Job interview	3.69 (1.01)	4.56 (1.03)	−3.42	15	0.004
**Heart rate (bpm)**						
Neutral	Blind date	77.2 (11.13)	81.8 (11.29)	−5.7	15	<0.001
Neutral	Job interview	77.2 (11.13)	84.2 (11.02)	−5.72	15	<0.001
Blind date	Job interview	81.8 (11.29)	84.2 (11.03)	−2.7	15	0.016

a Mean and standard deviation of condition 1.

b Mean and standard deviation of condition 2.

## Discussion

The goal of this experiment was to investigate whether exposure to various social scenes in virtual reality is associated with different levels of anxiety (H1). Our results based on subjective as well as objective measurements of anxiety yielded that as the social scene changed from the neutral VR world to the blind date scene or the job interview scene, the participants’ anxiety level significantly increased, which suggests that these various VR scenes could evoke different levels of anxiety. This indicates that virtual environments involving interactions between participants and virtual human (avatars) can be used to elicit anxiety among this group of participants.

This first study showed that our virtual social worlds can be used successfully to provoke levels of anxiety. In order to examine the association between different characteristics of the content of the dialogue and levels of social anxiety, the second study was conducted.

## Method (Second study: Dialogue Stressor Experiment)

### Ethics Statement

The second study was approved by Delft University of Technology Human Research Ethics Committee. Prior to the experiment, written informed consent was obtained from all participants, which also included their agreement to publish their (dialogue interaction) data with avatars in an anonymous fashion. After the experiment, participants received a chocolate bar and drink (tea, coffee or warm chocolate) as a token of appreciation for their participation.

### Dialogue Stressor

The social scene selected for the second experiment was a job interview, which consisted of a question and answer session. This VR selection was based on the first experiment result, where the job interview VR elicited more anxiety compared to the blind date VR. Each of dialogue unit in the job interview VR was defined as: [**virtual human’s question**] → [**participant’s answer**] → [**virtual human’s response comment**]. Each unit started with the virtual human posing a question related to the vacancy for which the participant had applied. The next block in the unit was the participant’s answer to this question, which was followed by the last unit block, the virtual human’s comment on that answer.

In order to influence anxiety levels, this dialogue unit could either be positive or negative. For example, the positive dialogue style consisted of a set of friendly, positive tone and enthusiastic type of questions and response comments, for example “*Why do you want to work for our company?*” and ended with the virtual human comment “*A very good reason indeed! I can see and feel a lot of passion in you!*” in reaction to the participant’s answer. On the other hand, the negative dialogue unit consisted of a set of unfriendly, negative tone, unenthusiastic and critical types of questions and responses, for example, “*Are you sure you want to work for our company?*” and ended with a virtual human’s comment “*That’s all? Nothing else? Seems as though you’re not so serious about working here!*”. A more comprehensive example of a typical dialogue interaction between a participant and an avatar as observed in the experiment is shown in the supporting information document (Text S1).

A dialogue database was created with a set of 95 positive dialogue units and a set of 95 negative dialogue units. The hypothesis was that the ratio by which an individual was exposed to a negative, instead of a positive dialogue unit, is correlated with his or her anxiety scores. Stated differently, it was expected that a dialogue stressor can induce anxiety levels.

### Experiment Design

**Figure 3:**
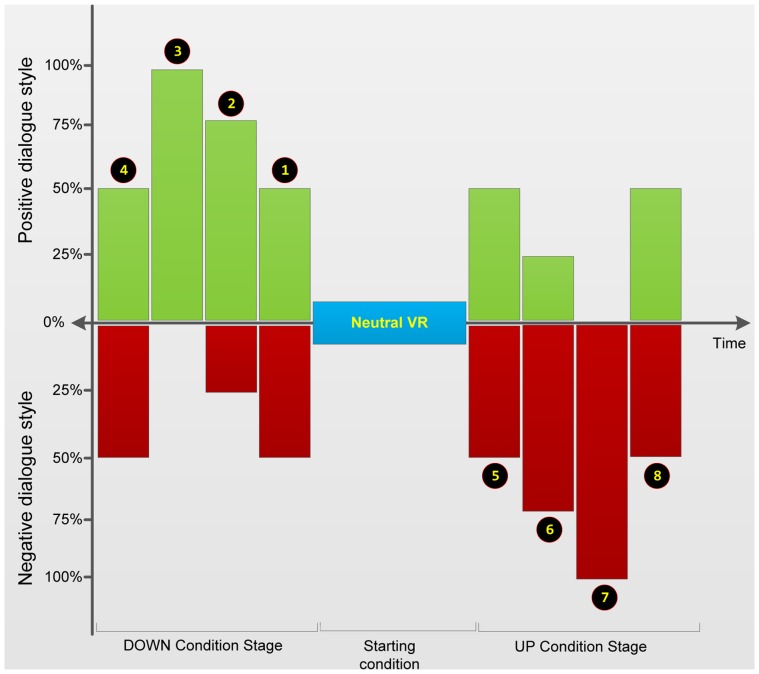
Two conditions that consist of eight dialogue stressor sub-conditions in the experiment.

The experiment was set up as a within-subjects design. All participants were exposed to 8 dialogue stressor sub-conditions (illustrated in Figure 3), divided into two main conditions: positive condition and negative condition. These sub-conditions were created by manipulating the ratio of positive and negative dialogue units to which individuals were exposed to in the job interview. In the positive condition, the experiment started with a 50% negative −50% positive dialogue ratio slot (C1) then continued to 25% negative −75% positive dialogue ratio slot (C2), 0% negative −100% positive dialogue ratio slot (C3) and going down again to 50% negative −50% positive dialogue ratio slot (C4). On the other hand, in the negative condition, the experiment started with a 50% negative −50% positive dialogue ratio slot (C5) then continued to 75% negative −25% positive dialogue ratio slot (C6), 100% negative −0% positive dialogue ratio slot (C7) and going up again to 50% negative −50% positive dialogue ratio slot (C8). Each slot lasted four minutes.

The negative condition consisted of more negative dialogue units in the conversations and resulted in a gradual increase of the dialogue stressor to the maximum level (start from C5 to C7), whereas the positive condition produced more positive dialogue units in the conversations that yielded a gradual decrease of the dialogue stressor to the minimum level (start from C1 to C3). The control conditions (C4 and C8) aimed to bring the dialogue stressor levels (whether it was maximum or minimum stressor levels) back to the starting conditions. The aim of these two conditions was to evaluate whether it was possible to control the level of anxiety variables from minimum or maximum stressor level and return the anxiety back to the original state (H3), suggesting extensive control of the phobic stressor.

In order to create specific ratio percentage of dialogue unit slots, the virtual database was created during the *run-time* by the system, hence each participant had 5 set of virtual databases (50% negative dialogue −50% positive dialogue, 25% negative dialogue −75% positive dialogue, 0% negative dialogue –100% positive dialogue, 75% negative dialogue –25% positive dialogue and 100% negative dialogue –0% positive dialogue). To make sure that participants were not answering identical question over and over again in the different dialogue conditions, a *question-index pointer* algorithm was used. This algorithm notified the dialogue system to select another question if the question has already been asked in the previous conditions.

**Figure 4:**
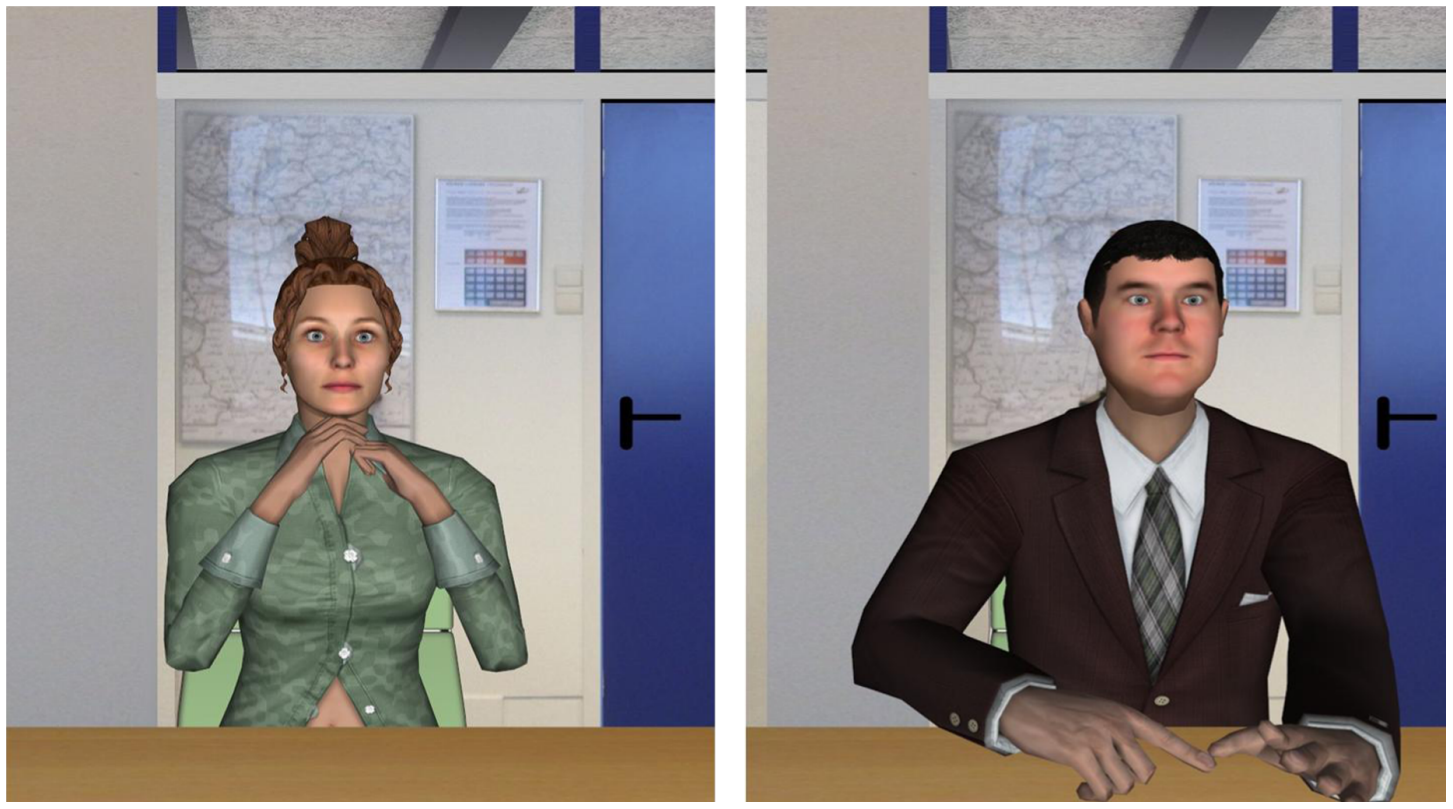
Virtual job interview with female (left) and male (right) interviewer.

To ensure impartiality and to avoid observer bias, the experiment was conducted in a double-blind mode. This meant that neither the participants nor the researcher who remotely controlled the virtual humans’ dialogue knew the critical aspects of the experiment (i.e. the dialogue stressor conditions). Furthermore the researcher could also not hear the virtual human as he was in another room where he could only hear the voice of participants over a speaker. The task of the researcher was to click on the questions/comments button when the participants finished their answer. The order of two main conditions and the assignment of the two virtual job interviewers (Figure 4) were counter-balanced.

### Participants

Twenty-four participants (13 females and 11 males) were recruited in the study that was approved by the ethics committee of Delft University of Technology. The age of participants ranged from 23 to 37 years (*M* = 29.37, *SD* = 3.28). The sample consisted of master and PhD students. Furthermore, all participants had seen 3D stereoscopic images or movies, but only five of them had ever used a virtual reality system before. Hence, all participants were naïve with respect to the hypotheses.

### Measurements

#### SUD Score

The levels of anxiety in the virtual environments in this study were again measured with the Subjective Units of Discomfort (SUD) [43].

#### Presence

Sense of being presence in the virtual reality environment was assessed with the Igroup Presence Questionnaire (IPQ) [44].

#### Heart rate

The heart rate was recorded with a Mobi8 device from TMSI with an Xpod Oximeter. The participants inserted their finger again into an adult articulated finger sensor.

#### Social Interaction Anxiety Scale (SIAS)

The SIAS [46] was used to evaluate social interactional anxiety and divide participants into groups of higher or lower social anxiety. The SIAS is a 20 items measure on which respondents rate their experiences in social situations associated with social anxiety. The items are rated on a 5-point scale from 0 (“not at all characteristic or true of me”) to 4 (“extremely characteristic or true of me”). The SIAS has been shown to be a reliable measurement of social anxiety [47].

#### Interview Attitude Questionnaire (IAQ)

The IAQ was designed for use in the current study in order to measure participants’ attitudes towards the job interview in the virtual reality. The IAQ is composed of the following six items that were rated on a seven-point Likert scale (from 1 to 7): pleasant – unpleasant (reversed), not relaxed – relaxed, aggressive – not aggressive, uncomfortable – comfortable, polite – impolite (reversed), and energizing – exhausting (reversed). The total IAQ score was calculated by adding up the (reversed) individuals items.

#### Dialogue Experience Questionnaire (DEQ)

To measure participants’ perception of the dialogue quality, e.g. flow, interaction and reality with the virtual human, the DEQ [37] was used. The total DEQ score was calculated by adding up items that were scored on 7-point Likert scale ranging from strongly disagree (1) to strongly agree (7).

#### Participant’s own Emotions and Perception of Virtual Human

The Self-Assessment Manikin (SAM) questionnaire was used to measure the participants’ own emotion and how they perceived the virtual human’s emotion during exposure in virtual reality. The SAM is a non-verbal pictorial assessment technique that measures the amount of pleasure, arousal, and dominance associated with a person’s affective reaction to a wide variety of stimuli [48]. Participants rated the dimensions of valence (positive-negative), arousal (excited-calm), and dominance (controlled-in control) via a pencil and paper version. For this experiment, the five-Likert scale manikin figures were taken from PXLab [49].

#### Verbal Communication Effect (Behavioural Changes)

Patients’ speech behavioural changes toward anxiety-provoking situations have been suggested as a variable to determine the level of distress or avoidance behaviour [11], [50]. This experiment used an auto-detect speech algorithm [51] to record the total time a participant spoke (in the unit of seconds) during a dialogue slot.

### Procedure and Apparatus

At the start of the experiment participants received a short introduction about the overall aim of the study and signed a consent form. After this, they completed the SIAS and the basic information questionnaire. The main part of the experiment consisted out of three sessions in the virtual reality world; participant started with exposure in the neutral VR world, after this they were exposed to two job interviews.

To get a baseline measurement and to familiarize the participants with virtual reality, they were exposed to the neutral VR world. This session lasted three minutes. In the other conditions, participants had 16 minutes of active free speech interactions (question – answer session related to the job position for which they had applied) with a virtual interviewer. This session was divided into four slots (Figure 3), which consisted of four minutes each and at the end of each slot participants were asked to give a SUD score. At the end of the two interview sessions, participants were asked to complete questionnaires related to the situation that they just experienced i.e. IAQ, DEQ and SAM questionnaires. After these two job interview sessions, participants filled in the IPQ questionnaire. The entire experiment took about 45 minutes.

All participants were exposed to a virtual reality environment using the DRVRET system with the same hardware configuration as the first study (the social scene experiment). The only difference was the HMD; in this experiment a Sony HMZ-T1 HMD was used. This HMD has 1200×720 pixels resolutions with 45 degrees field of view and was equipped with a custom-built head tracker using a triaxial gyroscope, an accelerometer and a compass sensors tracked at 6 MHz update rate.

## Results

In order to analyse the data (experiment data available at http://dx.doi.org/10.6084/m9.figshare.902844), a series of multivariate and univariate analyses were conducted. Where the sphericity assumption was violated a Greenhouse-Geisser correction was applied. To control for possible inflations of Type I Error, *post-hoc* analyses were conducted with Sidak correction.

### Low and High Social Anxiety Group

To examine the effect of the dialogue stressor on individuals with less or more social anxiety, the sample was split into two groups; a lower and a higher social anxiety group. These two groups were created based on the SIAS’s overall data (*M* = 24.9, *SD* = 12.6). Participants who scored below the mean score were assigned to the lower social anxiety group, while the other participants were assigned to the higher social anxiety group.

### Presence

Before performing further analyses on the effect of the dialogue stressor on the anxiety level, the reported presence level was analysed. The overall IPQ score was compared to the online IPQ data set (downloaded on February 09^th^, 2013. For comparison data see: http://www.igroup.org/pq/ipq/data.php) for stereo HMD visual stimuli. The overall IPQ rating (*M* = 50.17, *SD* = 5.35) in this experiment was significantly higher (*t*(59) =  −3.25, *p* = 0.002) than the overall IPQ online data set (*M* = 38.16, *SD* = 17.53), which suggests that participants in this study were more immersed than the presence level reported in other virtual world. Furthermore, there was no significant difference (*t*(22) = 0.35, p = 0.733) found between higher and lower social anxiety groups on the overall level of presence.

### Anxiety Level

In order to test the overall effect of the dialogue stressor conditions on the anxiety level, a doubly MANOVA repeated-measures was conducted, with dialog stressor (C1 to C8) as the independent within-subjects variables and the two social anxiety groups as between-subjects variable. The SUD score, heart rate and audio length were used as dependent variables. The results showed a significant overall main effect of dialogue stressor on anxiety level, (*F*(18,5) = 80.14, *p*<0.001, *η^2^* = 0.99). Furthermore, univariate analyses (see Table 2) showed significant effects of dialogue stressor on the SUD score, heart rate and audio length. These results confirm our hypothesis that dialogue stressor can impact anxiety levels (H2).

**Table 2:** Results of univariate analyses with dialogue stressor as within-subjects factor and social anxiety group as between-subjects factor on SUD score, heart rate and audio length.

Factor	Hyp. *df*	Error *df*	*F*	*p*	*η^2^*
**SUD score (0–10)**					
Dialogue stressors	2.99	65.52	28.57	<0.001	0.57
Social anxiety group (high and low)	1	22	8.72	0.007	0.28
Dialogue stressors**×**Social anxiety group	2.98	65.52	4.04	0.011	0.16
**Heart rate**					
Dialogue stressor	1.27	27.85	52.75	<0.001	0.71
Social anxiety group (high and low)	1	22	2.61	0.121	0.11
Dialogue stressors**×**Social anxiety group	1.27	27.85	4.14	0.043	0.16
**Audio length**					
Dialogue stressors	2.87	63.07	168.07	<0.001	0.88
Social anxiety group (high and low)	1	22	7.24	0.013	0.25
Dialogue stressors**×**Social anxiety group	2.87	63.07	1.30	0.281	0.06

*A post-hoc* test with Sidak correction was performed comparing SUD score, heart rate and audio length in all eight dialogue stressor conditions. The results are presented in Table 3. The *post-hoc* results showed that the participants’ overall anxiety level in virtual reality can be controlled up and down *on-the-fly* (dynamically) by using the dialogue stressor ratio combination. Furthermore, the correlations between on the one hand the dialogue stressor ratio and on the other hand the means of SUD score, heart rate and audio length in the eight dialogue conditions show a strong relationship: *r*(6) = 0.91, *p* = 0.002; *r*(6) = 0.76, *p* = 0.028 and *r*(6) =  −0.94, *p* = 0.001 respectively.

**Table 3:** Comparison between dialog stressor on SUD score rating, heart rate (bpm) and audio length (second).

Measurement		*M1*(*SD*)^e^	*M2*(*SD*)^f^	*t*	*df*	*p*
Condition 1	Condition 2					
**SUD score (0–10)**						
50% (end)^a^	0% (C4)^d^	3.88 (0.74)	3.04 (1.27)	−3.75	23	0.001
0%	25%	3.04 (1.27)	3.67 (0.92)	3.32	23	0.003
25%	50% (avg.)^b^	3.67 (0.92)	3.63 (0.84)	0.28	23	0.784
50% (avg.)^b^	75%	3.63 (0.84)	4.42 (1.1)	5.75	23	<0.001
75%	100%	4.42 (1.1)	5.42 (1.1)	−5.54	23	<0.001
100%	50% (end)^c^ (C8)^d^	5.42 (1.1)	4.25 (1.26)	8.14	23	<0.001
**Heart rate (bpm)**						
50% (end)^a^	0% (C4)^d^	82.6 (4.7)	82.3 (3.7)	0.69	23	0.499
0%	25%	82.3 (3.7)	84.5 (5.4)	4.16	23	<0.001
25%	50% (avg.)^b^	84.5 (5.4)	85.8 (7.1)	−2.98	23	0.007
50% (avg.)^b^	75%	85.8 (7.1)	90.2 (9.7)	6.83	23	<0.001
75%	100%	90.2 (9.7)	92.6 (10.2)	−8.84	23	<0.001
100%	50% (end)^c^ (C8)^d^	92.6 (10.2)	92.5 (9.9)	0.46	23	0.647
**Audio Length (s)**						
50% (end)^a^	0% (C4)^d^	134 (25.9)	177.7 (21.6)	9.7	23	<0.001
0%	25%	177.7 (21.6)	120 (13)	−16.68	23	<0.001
25%	50% (avg.)^b^	120 (13)	101.6 (14.1)	7.06	23	<0.001
50% (avg.)^b^	75%	101.6 (14.1)	71.3 (19)	−10.92	23	<0.001
75%	100%	71.3 (19)	50.6 (13.8)	6.8	23	<0.001
100%	50% (end)c (C8)^d^	50.6 (13.8)	82.7 (16.3)	−10.94	23	<0.001

a Value from the last 50% dialog stressor in the positive condition (C4).

b Average value from the first 50% dialog stressor in both negative and positive condition (C1&C5).

c Value from the last 50% dialogue stressor in the negative condition (C8).

d The control conditions.

e Mean and standard deviation of condition 1.

f Mean and standard deviation of condition 2.

**Figure 5:**
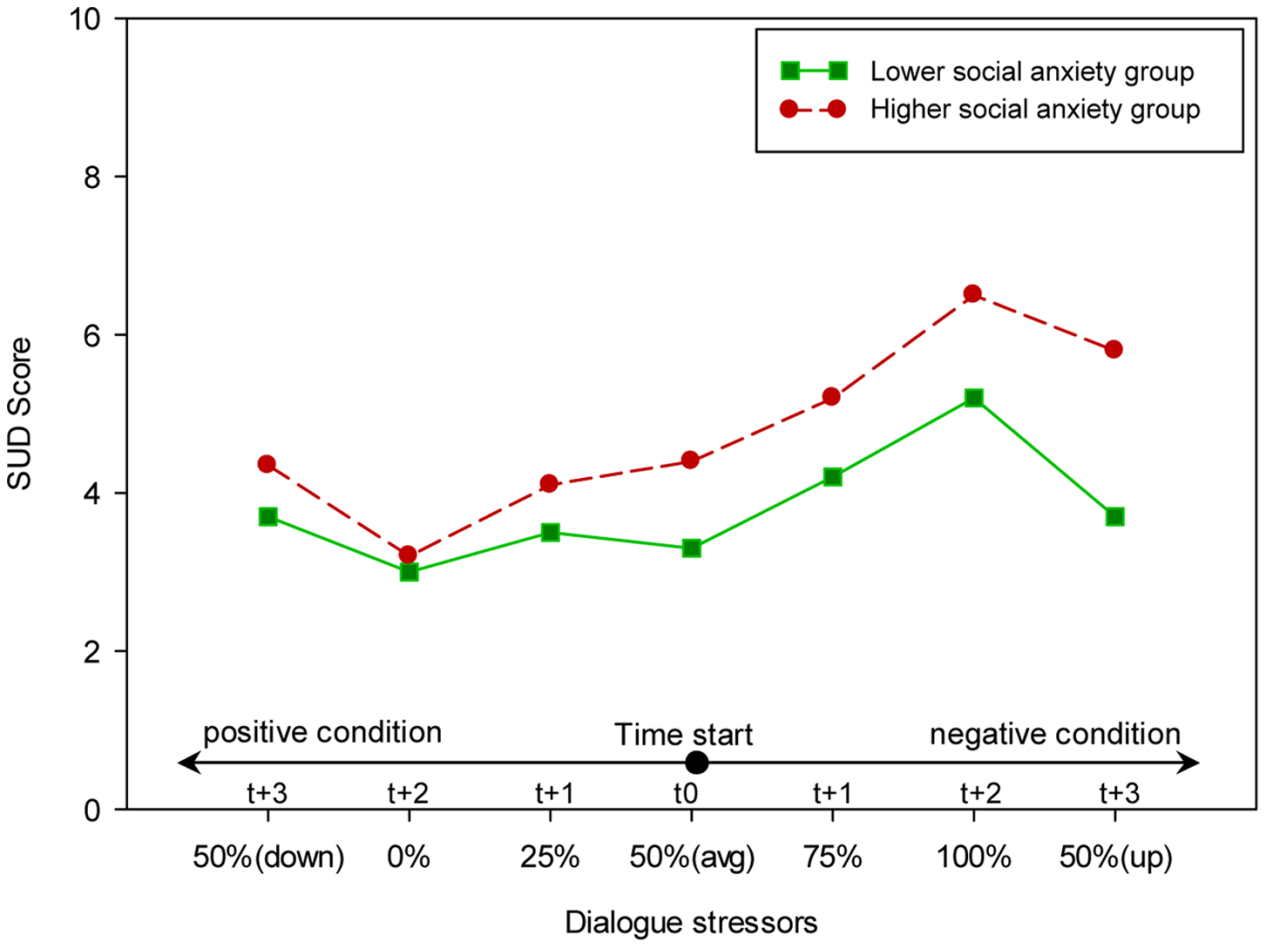
The effect of dialogues stressor on the participants’ SUD score.

**Figure 6:**
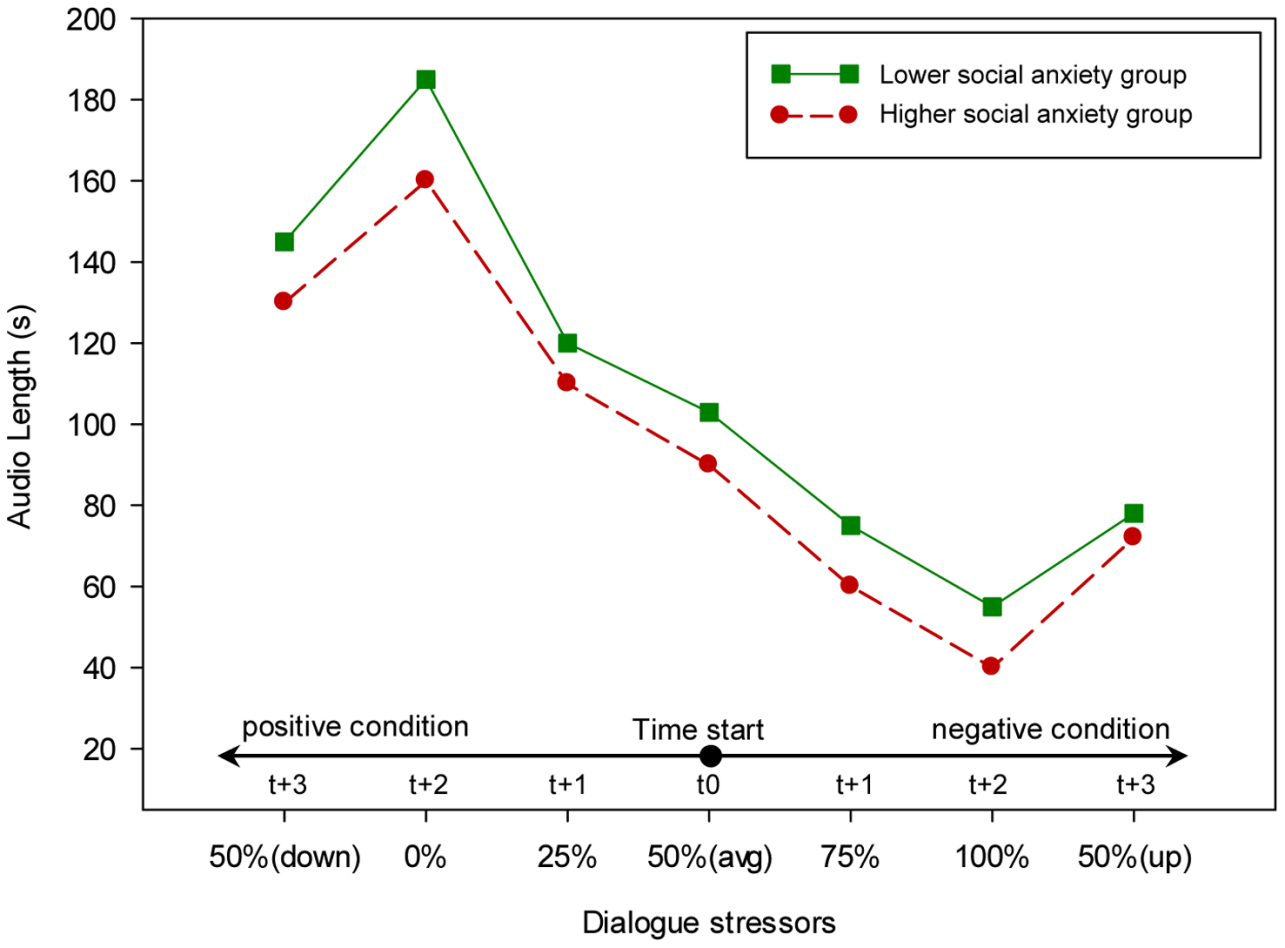
The effect of dialogues stressor on the participants’ verbal communication (the length of the speak).

The result of the overall analysis also showed that there was a significant overall main effect for the higher and lower social anxiety groups, (*F*(3,20) = 3.25, *p* = 0.044, *η^2^* = 0.33). Further, the univariate test (see Table 2) showed a significant difference between the two groups on the SUD Score rating and audio length. As shown in Figure 5 and Figure 6, the higher social anxiety group rated the SUD score significantly higher and spoke significantly less than the lower social anxiety group. However, no significant difference between the higher and lower social anxiety groups was found regarding heart rate.

**Figure 7:**
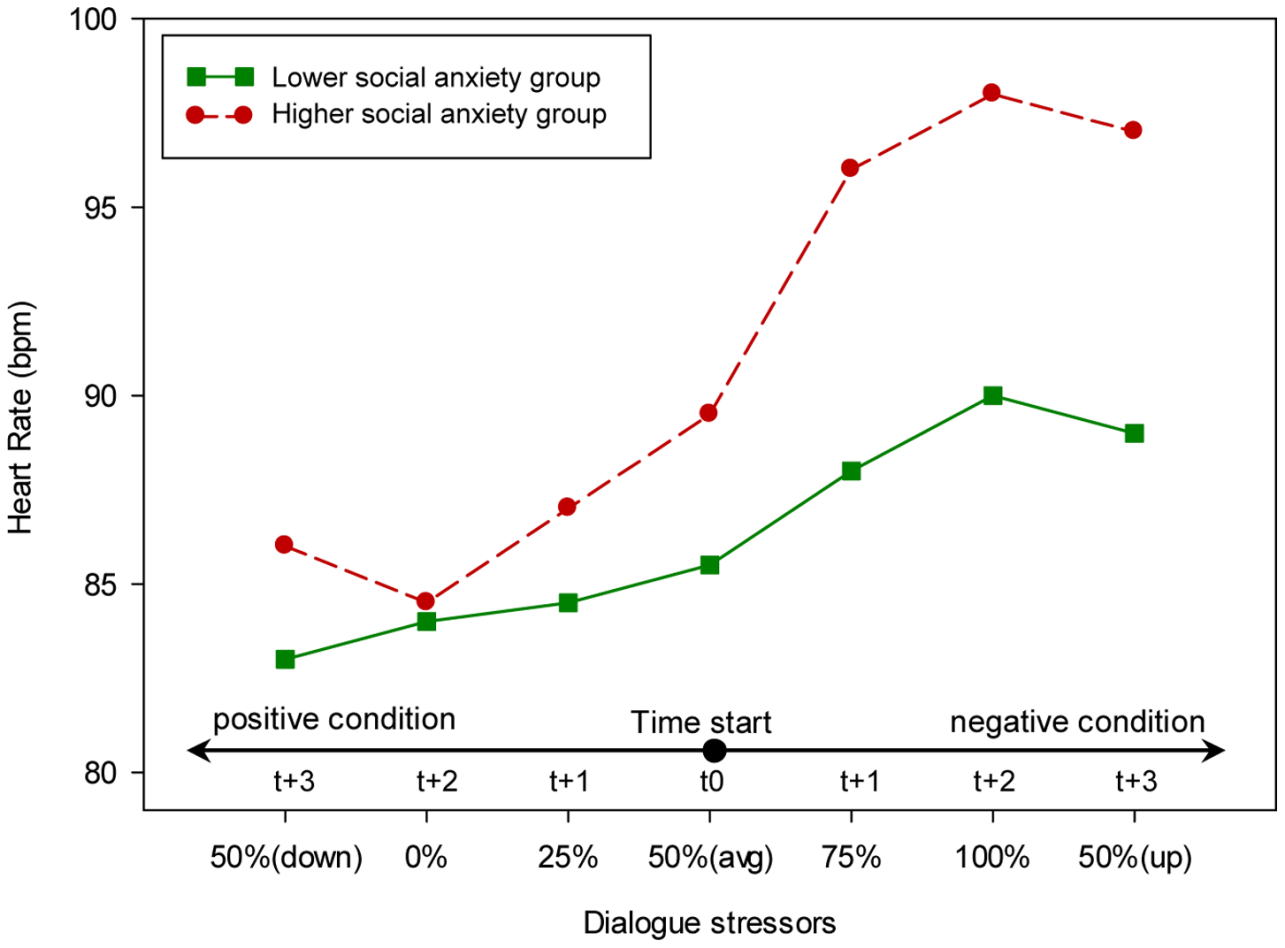
The effect of dialogues stressor on the participants’ heart rate.

The overall doubly repeated-measure MANOVA found no significant overall two-way interaction effect between dialogue stressor and the two social anxiety groups on anxiety level, (*F*(18,5) = 2.14, *p* = 0.205, *η^2^* = 0.89). However, additional univariate analyses found a significant two-way interaction effect between dialogue stressor and the two social anxiety groups on SUD score rating and heart rate, as shown in Table 2. Furthermore, and as can be seen in Figure 5 and Figure 7, the difference between the higher and lower social anxiety groups in the SUD score rating and heart rate varied across conditions. For example, the higher social anxiety group reported a significantly higher (*t*(22) =  −3.35, *p* = 0.003) SUD score rating in the maximum level of the dialogue stressor condition (100% negative dialogue style ratio) while no significant difference (*t*(22) = 0.27, *p* = 0.79) was found in the minimum level of the dialogue stressor condition (0% negative dialogue style ratio) between the two social anxiety groups. A similar pattern was found in heart rate. As can be seen in Figure 6, the higher social anxiety group showed a significantly higher (*t*(22) =  −1.79, *p* = 0.048) heart rate in the maximum level of dialogue stressor condition (100% negative dialogue style ratio) while no significant difference (*t*(22) = 0.42, *p* = 0.68) was found in the minimum level of dialogue stressor condition (0% negative dialogue style ratio) between the two social anxiety groups. Furthermore, no significant two-way interaction effect was found between different dialogue stressor and the two groups on the audio length, which seem to have a fixed difference between the two groups across the eight conditions.

As reported above, this experiment applied two control conditions (C4 and C8) (see also Figure 3). The C4 control condition aimed to bring the minimum dialog stressor levels (C3) back up to the starting conditions (C1), whilst the C8 control condition aimed to bring the maximum dialog stressor levels (C7) back down to the starting conditions (C5). *Post-hoc* analysis (see Table 3) of both the SUD score rating and verbal dialogue time (audio length) confirmed that both control conditions were able to increase and decrease (subjective) anxiety levels significantly from the minimum and maximum dialogue stressor level. Although this seems to support the hypothesis that the level of anxiety can be controlled into some specific level after it stretches to the maximum/minimum specific level (H3), no significant difference for the control conditions were found in heart rate of the participant.

### Participants’ Emotion

For the participants’ emotional experience during the exposure, univariate analyses on the SAM rating found significant effects for two dialogue stressor conditions (C1–C4 and C5–C8) on the valence, arousal and dominance affective dimensions (see Table 4). Compared to the negative dialogue condition, in the positive dialogue condition participants rated their emotional state as significantly more pleasant, more excited and more dominant (see Table 5).

**Table 4:** Results of univariate analyses with dialogue stressor as within-subjects factor and social anxiety group as between-subjects factor on the individuals’ own valence, arousal and dominance state.

Factor	Hyp. *df*	Error *df*	*F*	*p*	*η^2^*
**Valence**					
Dialogue stressors	1	22	38.5	<0.001	0.68
Social anxiety group (high and low)	1	22	0.13	0.724	0.01
Dialogue stressors**×**Social anxiety group	1	22	7.07	0.014	0.24
**Arousal**					
Dialogue stressor	1	22	19.98	<0.001	0.48
Social anxiety group (high and low)	1	22	4.21	0.052	0.16
Dialogue stressors**×**Social anxiety group	1	22	9.09	0.006	0.29
**Dominance**					
Dialogue stressors	1	22	32.2	<0.001	0.6
Social anxiety group (high and low)	1	22	8.75	0.007	0.29
Dialogue stressors**×**Social anxiety group	1	22	4.37	0.048	0.17

**Table 5:** Comparison between different conditions on the individuals’ own valence, arousal and dominance state.

Measurement	*M(SD)* Negative condition	*M(SD)* Positive condition	*t*	*df*	*p*
Valence	2.63 (0.88)	4.00 (0.78)	−5.01	23	<0.001
Arousal	2.67 (0.76)	3.46 (0.78)	−2.94	23	0.007
Dominance	3.17 (0.92)	4.46 (0.59)	−4.99	23	<0.001

**Figure 8:**
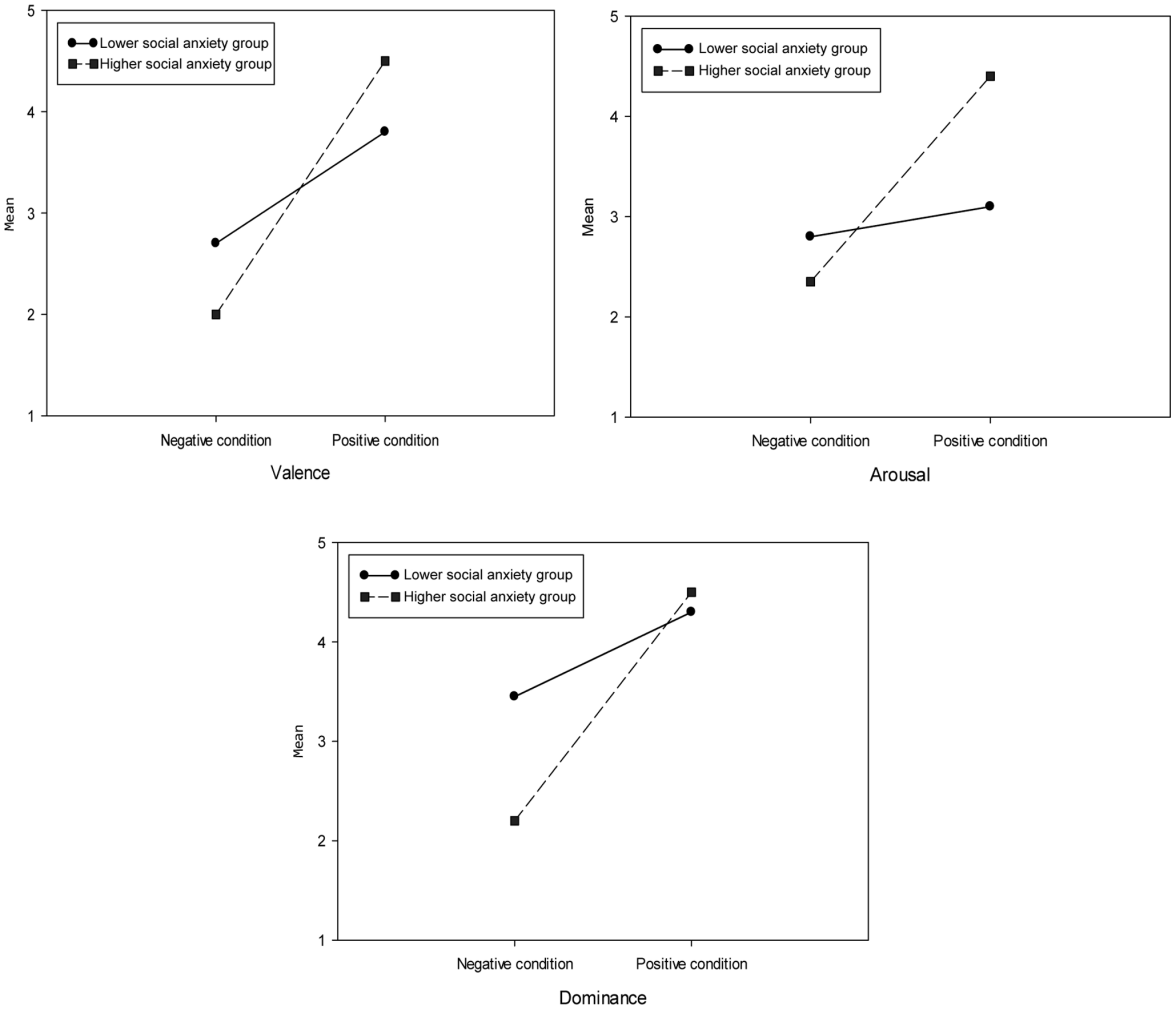
The effect of dialogue stressor on participants’ own emotion in three affective dimensions.

Univariate analyses also found a significant two-way interaction effect for the anxiety groups and the dialogue stressor on the valence, arousal and dominance affective dimensions (see Table 4). As depicted in Figure 8, on the valence dimension, the higher social anxiety group rated valence significantly higher (*t*(22) = 1.32, *p* = 0.026) than the lower social anxiety group in the positive condition, while no significant difference (*t*(22) = 0.24, *p* = 0.062) was found in the negative condition between the two groups. On the arousal dimension, the high social anxiety group reported significantly more (*t*(22) = 4.18, *p* = 0.003) arousal than the low social anxiety group in the positive condition, while again no significant difference (*t*(22) = 0.13, *p* = 0.085) was found in the negative condition. Finally for the dominance affective dimension, the higher social anxiety group felt significantly less (*t*(22) = 5.72, *p* = 0.001) dominant than the lower social anxiety group in the negative condition, while this time no significant difference (*t*(22) = 0.2, *p* = 0.097) was found in the positive condition. It seems therefore that the higher social anxiety group was more affected by dialogue stressor, while the lower social anxiety group was more stable across the positive and negative conditions.

### Perception of Virtual human’s’ Emotion

Univariate analyses on the SAM rating of participants’ perceptions of the virtual human’s emotion (see Table 6) showed a significant effect for the dialogue stressor on three affective dimensions. Compared to the negative dialogue condition, the virtual human in the positive dialogue condition was perceived to be in state of significantly higher valence and arousal, but significantly lower dominance (see Table 7).

**Table 6:** Results of univariate analyses with dialogue stressor as within-subjects factor and social anxiety group as between-subjects factor on perceived valence, arousal and dominance of the virtual human.

Factor	Hyp. *df*	Error *df*	*F*	*p*	*η^2^*
**Valence**					
Dialogue stressors	1	22	166.91	<0.001	0.88
Social anxiety group (high and low)	1	22	1.71	0.205	0.07
Dialogue stressors**×**Social anxiety group	1	22	14.07	0.001	0.39
**Arousal**					
Dialogue stressor	1	22	10.64	0.004	0.33
Social anxiety group (high and low)	1	22	4.97	0.036	0.18
Dialogue stressors**×**Social anxiety group	1	22	3.2	0.088	0.13
**Dominance**					
Dialogue stressors	1	22	25.27	<0.001	0.54
Social anxiety group (high and low)	1	22	4.13	0.055	0.16
Dialogue stressors**×**Social anxiety group	1	22	0.52	0.48	0.23

**Table 7:** Comparison between different conditions on perceived valence, arousal and dominance of the virtual human.

Measurement	*M(SD)* Negative condition	*M(SD)* Positive condition	*t*	*df*	*p*
Valence	1.71 (0.62)	3.92 (0.72)	−10.18	23	<0.001
Arousal	3.04 (0.62)	3.67 (0.82)	−2.61	23	0.016
Dominance	4.1 (0.76)	3.08 (0.71)	5.45	23	<0.001

**Figure 9:**
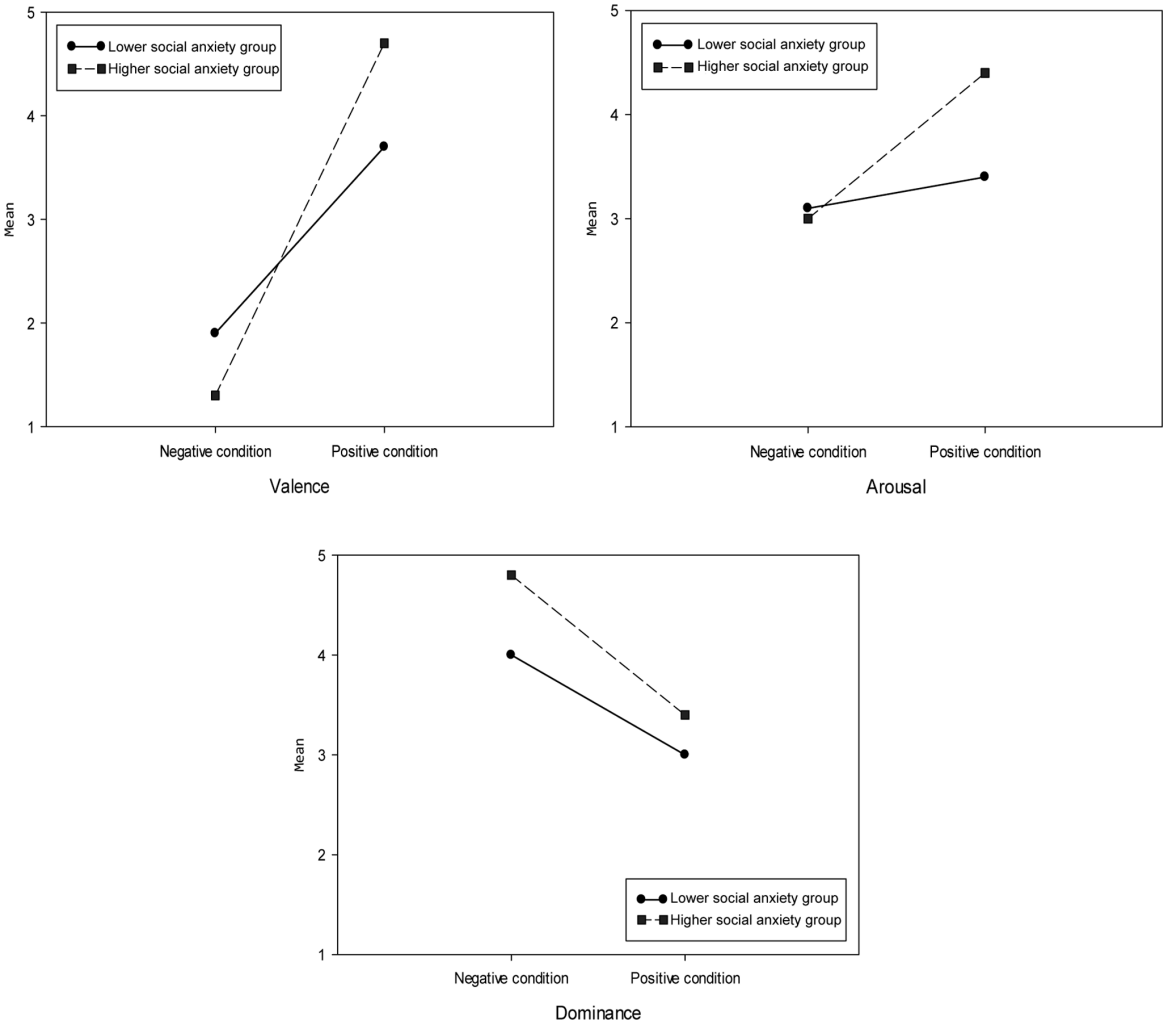
The effect of dialogue stressor on virtual human’s emotion in three affective dimensions.

A two-way effect for dialogue stressor and anxiety groups was only found on the valence (*F*(1,22) = 14.07, *p* = 0.001, *η^2^* = 0.39) affective dimension. As Figure 9 on valence rating shows, compared to the lower social anxiety group (*M* = 3.67, *SD* = 0.59), participants in the higher social anxiety group (*M* = 4.67, *SD* = 0.52) perceived the virtual human as exhibiting significantly more (*t*(22) =  −2.58, *p* = 0.017) arousal. No significant difference (*t*(22) = 0.19, *p* = 0.8) however was found in the negative condition between the two groups.

### Dialog Experience and Interview Attitude

**Figure 10:**
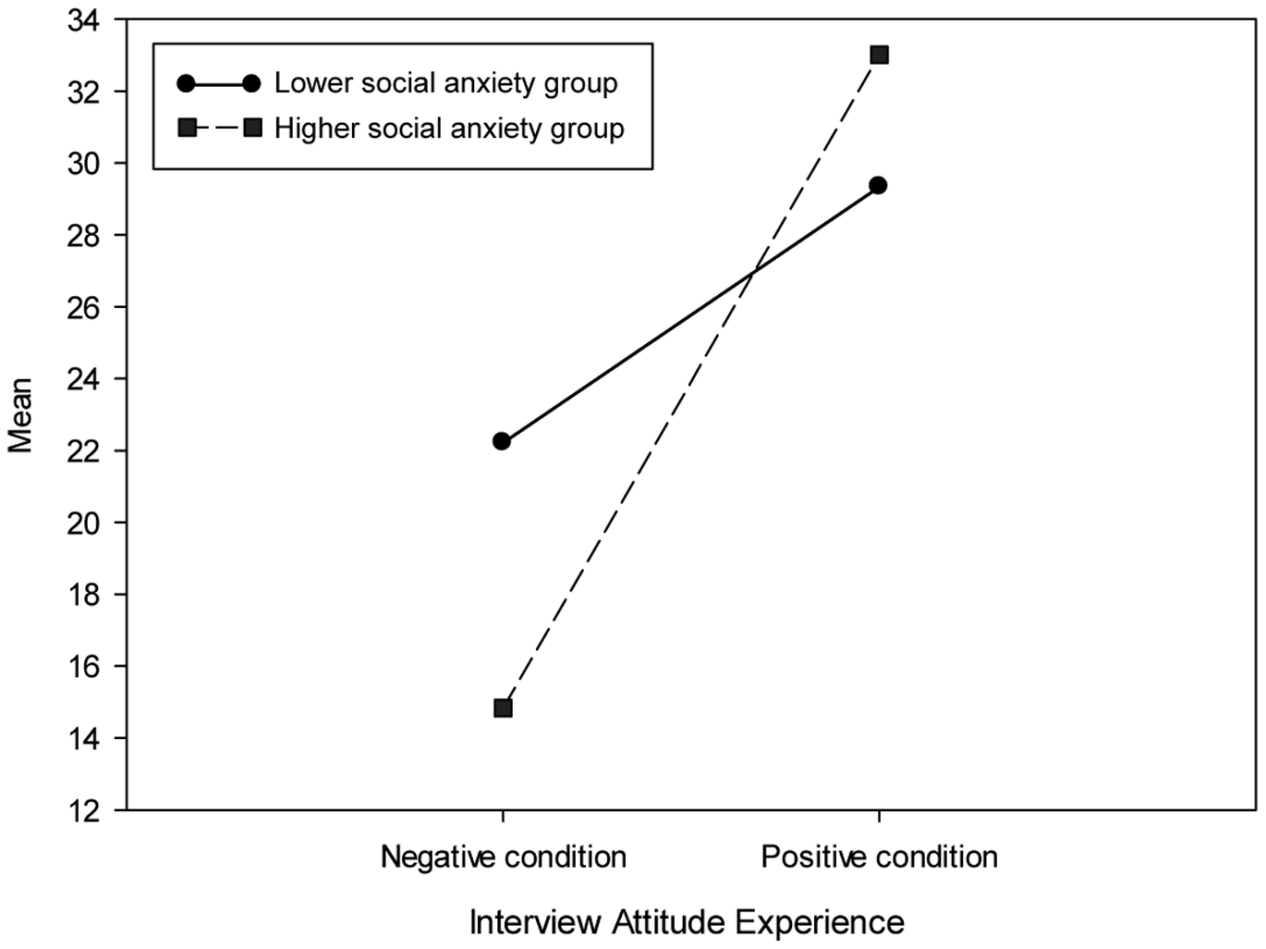
The effect of dialogue stressor on participant’s interview attitude experience.

Analysis of the total DEQ score and IAQ score showed that compared to the negative dialogue condition (DEQ: *M* = 32.84, *SD* = 0.99; IAQ: *M* = 20.36, *SD* = 4.2), in the positive condition (DEQ: *M* = 38.33, *SD = *1.24; IAQ: *M* = 30.25, *SD* = 3.21) participants rated their dialogue experience more positively (*F*(1,22) = 252.56, *p*<0.001) and they also had a significantly (*F*(1,22) = 103.6, *p*<0.001) more positive attitude toward the interview. On average the higher social anxiety group (*M* = 23.92, *SD* = 0.92) rated their attitude significant lower (*F*(1,22) = 22.38, *p*<0.001) than participants in the lower social anxiety group (*M* = 25.78, *SD* = 0.81). The analysis of the IAQ score also revealed a significant two-way interaction effect (*F*(1,22) = 19.82, *p*<0.001) for the anxiety group and dialogue stressor. As Figure 10 shows, in the negative dialogue condition, the attitude of the higher social anxiety group was significantly lower (*t*(22) = 3.55, *p* = 0.015) than the lower social anxiety group, while in the positive dialogue condition there was no significant difference (*t*(22) =  −1.85, *p* = 0.12) between both groups. Moreover, the lower social anxiety group rated their attitude significantly more positively (*t*(17) =  −10.16, *p*<0.001) in the positive dialogue condition compared to the negative dialogue condition. Likewise, the higher social anxiety group rated their attitude significantly more positively (*t*(5) =  −4.64, *p* = 0.006) in the positive dialogue condition compared to the negative dialogue condition.

## Discussion and Conclusion

The results of the first study support the hypothesis that various social scenes in virtual reality can have a different effect on the individuals’ anxiety levels. As the social situation changed from a neutral VR world to a blind date situation or a job interview situation, the individuals’ anxiety level significantly increased, which suggests that various VR situations can evoke different levels of anxiety. Furthermore, in the second study, the manipulation of a dialogue feedback stressor in the dialogue-based VRET system for social anxiety had a significant effect on individuals’ level of anxiety, their attitude, their dialogue experiences, their speech behaviour, their own emotions, and how they perceived the emotion of the virtual human. These results suggest that within a dialogue the ratio of positive and negative responses can be applied as an effective anxiety stressor. Of importance are also the results found in the control condition (C4 and C8) where polarity of ratio was again reversed. This showed that levels of anxiety could be influenced and controlled dynamically; i.e. anxiety could not only be increased or decreased to a maximum or a minimum level, but also reversed halfway. This is important as it would give therapists extensive freedom to stabilize anxiety of a person on a desired level. Finally, participants with more social anxiety were more affected by the dialogue stressor than other participants. Our results show that the individuals’ degree of social anxiety is related to the level of anxiety and avoidance behaviour, i.e. less speaking during exposure. This was also related to how they perceived the emotion of the virtual human and reported on their own emotion.

Our findings have implications with regard to virtual reality exposure therapy for anxiety disorders in general and social anxiety disorder in particular. First and foremost, our results indicate that virtual social interactions can be effectively used in virtual reality exposure therapy to provoke social anxiety. Additionally, our results demonstrate that social dialogues in virtual environments can be effectively manipulated for therapeutic purposes. Accordingly, our findings are promising with regard to the use of virtual reality exposure therapy for different psychological complaints that might involve social interactions between patients and virtual human. For example, virtual social environments might be effectively used to train social skills for individuals with other complaints than social phobia. Furthermore, this study builds on previous work that demonstrated that specific social scenes [29], [30], avatars’ body posture [52] and avatar’s facial expression [53] can elicit anxiety. This study adds to this body of work by introducing the dialogue stressor ratio that can be manipulated *on-the-fly* within the scene. Hence, it can give the therapist the flexibility to control social stressors during exposure.

Despite the promising results, the studies also have limitations that should be considered. First, the dialogue system was designed as such that the computer took the lead in the conversation in order to limit the number of potential answers by the participants. However, some social anxiety disorder patients might specifically fear a situation where they have to start and lead a conversation. They therefore might benefit from being exposed to such situations. Second, the social setting focused on two scenes only: blind date and job interview, where social phobia patients normally fear a range of social situations. Still, the findings might generalise to these other situations if they incorporate a question and answer style conversation. Third, this study did not include individuals diagnosed with social anxiety disorder. If anxiety is considered a continuous scale, the difference found in this study between lower and higher anxiety individuals might also be generalised to individuals higher up the social anxiety scale. Fourth, in the second study, the post self-reported experience of individuals’ own emotion showed a significant reduction in the arousal, valence, and dominance dimensions in the negative compared to the positive dialogue condition. This might suggest that participants experienced the emotion of hurt alongside or instead of anxiety following exposure. However, the self-reported subjective discomfort and heart rate data collected during exposure rather indicated an anxiety response during exposure. Future research should consider using multi-dimensional emotion measures [48], [54] during virtual reality exposure.

The main scientific contribution of the study lies in the validation of dialogue stressor as a stimulus that can induce anxiety. Besides psychotherapy, this could also benefit other application domains such as psychological stress testing, e.g. Trier Social Stress Test (TSST) training [55]–[57], job interview or negotiation simulations [58], or gaming. As in all these domains individuals could benefit from conversation that could elicit stress.

## Supporting Information

Text S1 **A typical dialogue interaction between a participant (P) and an avatar (A) as observed in the experiment.**
(DOCX)
