# Correction: Controlling Social Stress in Virtual Reality Environments

**DOI:** 10.1371/journal.pone.0223988

**Published:** 2019-10-11

**Authors:** Dwi Hartanto, Isabel L. Kampmann, Nexhmedin Morina, Paul G. M. Emmelkamp, Mark A. Neerincx, Willem-Paul Brinkman

As a result of a re-analysis of the statistical analyses reported in one of the authors’ draft PhD theses, which includes this article [1], the authors provide updates to the published article.

The corrections provided here address rounding errors, *t*-test with incorrect equal variance assumed, not applying post-hoc correction on *p*-values, inappropriate application of one-tailed *t*-test, errors in the reported degrees of freedom, *F*-values, and associated effect-size. Furthermore, the corrections also address textual errors in reference to figures and measures. To maintain transparency, the authors also provide the data files, data analysis scripts (open source R-scripts), and statistical output files for the re-analysis (available using DOI: https://doi.org/10.4121/uuid:030f90e6-c126-4bdd-81b4-eb65ddd6f2bc).

The corrected sentences are provided below. The corrections do not change the overall conclusions. Please see the location of the error, the original text, and the author-corrected text here.

**Table:** 

Location	Original text	Corrected text
Abstract, eighth sentence	“The correlations between on the one hand the dialogue stressor ratio and on the other hand the means of SUD score, heart rate and audio length in the eight dialogue conditions showed a strong relationship: *r*(6) = 0.91, *p* = 0.002; *r*(6) = 0.76, *p* = 0.028 and *r*(6) = −0.94, *p* = 0.001 respectively.”	“The correlations between on the one hand the dialogue stressor ratio and on the other hand the means of SUD score, heart rate and audio length in the eight dialogue conditions showed a strong relationship: *r*(6) = 0.90, *p* = 0.002; *r*(6) = 0.74, *p* = 0.036 and *r*(6) = -0.91, *p* = 0.002 respectively.”
Results, second paragraph, third sentence	“The overall IPQ rating (*M* = 52.44, *SD* = 3.05) in this experiment was significantly higher (*t*(51) = −3.22, *p* = 0.002) than the overall IPQ online data set (*M* = 38.16, *SD* = 17.53), which suggest that the participants were more immersed than the level reported in other virtual worlds.”	“The overall IPQ rating (*M* = 52.44, *SD* = 3.05) in this experiment was significantly higher (*t*(40.98) = −4.64, *p* < 0.001) than the overall IPQ online data set (*M* = 38.93, *SD* = 17.09), which suggests that the participants were more immersed than the level reported in other virtual worlds.”
Results, third paragraph, third and fourth sentences	“The results showed a significant effect of different virtual social scenes on anxiety levels, (*F*(4,12) = 16.94, *p*<0.001, *η*^*2*^ = 0.85). Furthermore, univariate analyses found significant effects (*F*(2,30) = 36.65, *p*<0.001, *η*^*2*^ = 0.71) on the SUD score and heart rate (*F*(2,30) = 23.52, *p*<0.001, *η*^*2*^ = 0.61).”	“The results showed a significant effect of different virtual social scenes on anxiety levels, (*F*(4,12) = 19.14, *p* < 0.001, *η*^2^ = 0.87). Furthermore, univariate analyses found significant effects (*F*(2,30) = 36.65, *p* < 0.001, *η*^2^ = 0.71) on the SUD score and heart rate (*F*(2,30) = 23.08, *p* < 0.001, *η*^2^ = 0.61).”
Method (Second study: Dialogue Stressor Experiment), Participants subsection, second sentence	“The age of participants ranged from 23 to 37 years (*M* = 29.37, *SD* = 3.28).”	“The age of participants ranged from 23 to 37 years (*M* = 29.38, *SD* = 3.28).”
Results, Low and High Social Anxiety Group subsection, second sentence	“These two groups were created based on the SIAS’s overall data (*M* = 24.9, *SD* = 12.6).”	“These two groups were created based on the SIAS’s overall data (*M* = 25.0, *SD* = 12.6).”
Results, Presence subsection, fourth sentence	“The overall IPQ rating (M = 50.17, SD = 5.35) in this experiment was significantly higher (t(59) = -3.25, p = 0.002) than the overall IPQ online data set (M = 38.16, SD = 17.53), which suggests that participants in this study were more immersed than the presence level reported in other virtual world.”	“The overall IPQ rating (*M* = 50.17, *SD* = 5.35) in this experiment was significantly higher (*t*(46.04) = 3.73, *p* < 0.001) than the overall IPQ online data set (*M* = 38.93, *SD* = 17.09), which suggests that participants in this study were more immersed than the presence level reported in other virtual world.”
Results, Anxiety Level subsection, first paragraph, third sentence	“The results showed a significant overall main effect of dialogue stressor on anxiety level, (*F*(18,5) = 80.14, *p*<0.001, *η*^*2*^ = 0.99).”	“The results showed a significant overall main effect of dialogue stressor on anxiety level, (*F*(18,5) = 106.01, *p* < 0.001, *η*^2^ = 1.00).”
Results, Anxiety Level subsection, second paragraph, fourth sentence	“Furthermore, the correlations between on the one hand the dialogue stressor ratio and on the other hand the means of SUD score, heart rate and audio length in the eight dialogue conditions show a strong relationship: *r*(6) = 0.91, *p* = 0.002; *r*(6) = 0.76, *p* = 0.028 and *r*(6) = −0.94, *p* = 0.001 respectively.”	“Furthermore, the correlations between on the one hand the dialogue stressor ratio and on the other hand the means of SUD score, heart rate and audio length in the eight dialogue conditions show a strong relationship: *r*(6) = 0.90, *p* = 0.002; *r*(6) = 0.74, *p* = 0.036 and *r*(6) = −0.91, *p* = 0.002 respectively.”
Results, Anxiety Level subsection, third paragraph, first sentence	“The result of the overall analysis also showed that there was a significant overall main effect for the higher and lower social anxiety groups, (*F*(3,20) = 3.25, *p* = 0.044, *η*^*2*^ = 0.33).”	“The result of the overall analysis showed that there was no significant overall main effect for the higher and lower social anxiety groups, (*F*(3,20) = 2.87, *p* = 0.062, *η*^2^ = 0.30). However,”
Results, Anxiety Level subsection, fourth paragraph, first sentence	“The overall doubly repeated-measure MANOVA found no significant overall two-way interaction effect between dialogue stressor and the two social anxiety groups on anxiety level, (*F*(18,5) = 2.14, *p* = 0.205, *η*^*2*^ = 0.89).”	“The overall doubly repeated-measure MANOVA found no significant overall two-way interaction effect between dialogue stressor and the two social anxiety groups on anxiety level, (*F*(18,5) = 2.34, *p* = 0.176, *η*^2^ = 0.89).”
Results, Anxiety Level subsection, fourth paragraph, fifth and sixth sentences	“A similar pattern was found in heart rate. As can be seen in Fig 6, the higher social anxiety group showed a significantly higher (*t*(22) = −1.79, *p* = 0.048) heart rate in the maximum level of dialogue stressor condition (100% negative dialogue style ratio) while no significant difference (*t*(22) = 0.42, *p* = 0.68) was found in the minimum level of dialogue stressor condition (0% negative dialogue style ratio) between the two social anxiety groups.”	“A similar pattern seems to appear in the heart rate at those two points in Fig 7, although not significant this time (100% negative dialogue style ratio: *t*(22) = −1.78, *p* = 0.088; 0% negative dialogue style ratio: *t*(5.53) = -0.28, *p* = 0.79).”
Results, Participants’ Emotion subsection, second paragraph, second through fourth sentences	“As depicted in Fig 8, on the valence dimension, the higher social anxiety group rated valence significantly higher (*t*(22) = 1.32, *p* = 0.026) than the lower social anxiety group in the positive condition, while no significant difference (*t*(22) = 0.24, *p* = 0.062) was found in the negative condition between the two groups. On the arousal dimension, the high social anxiety group reported significantly more (*t*(22) = 4.18, *p* = 0.003) arousal than the low social anxiety group in the positive condition, while again no significant difference (*t*(22) = 0.13, *p* = 0.085) was found in the negative condition. Finally for the dominance affective dimension, the higher social anxiety group felt significantly less (*t*(22) = 5.72, *p* = 0.001) dominant than the lower social anxiety group in the negative condition, while this time no significant difference (*t*(22) = 0.2, *p* = 0.097) was found in the positive condition.”	“As depicted in Fig 8, on the arousal dimension, the high social anxiety group reported significantly more (*t*(5.47) = -2.71, *p* = 0.039) arousal than the low social anxiety group in the positive condition, while no significant difference (*t*(22) = 1.25, *p* = 0.22) was found in the negative condition. Furthermore, for the dominance affective dimension, the higher social anxiety group felt significantly less (*t*(22) = 2.98, *p* = 0.007) dominant than the lower social anxiety group in the negative condition, while this time no significant difference (*t*(22) = -0.20, *p* = 0.846) was found in the positive condition. Finally for the valence dimension, the higher social anxiety group rated valence not significantly higher (*t*(22) = -1.91, *p* = 0.069) than the lower social anxiety group in the positive condition, and no significant difference (*t*(5.82) = 1.55, *p* = 0.173) was found in the negative condition between the two groups.”
Results, Perception of Virtual human’s’ Emotion sub-section, second paragraph, second and third sentences	“As Fig 9 on valence rating shows, compared to the lower social anxiety group (*M* = 3.67, *SD* = 0.59), participants in the higher social anxiety group (*M* = 4.67, *SD* = 0.52) perceived the virtual human as exhibiting significantly more (*t*(22) = −2.58, *p* = 0.017) arousal. No significant difference (*t*(22) = 0.19, *p* = 0.8) however was found in the negative condition between the two groups.”	“As Fig 9 on valence rating shows, compared to the lower social anxiety group (*M* = 3.67, *SD* = 0.59), participants in the higher social anxiety group (*M* = 4.67, *SD* = 0.52) perceived the virtual human as exhibiting significantly more (*t*(22) = −3.67, *p* = 0.001) valence. No significant difference (*t*(22) = 1.78, *p* = 0.89) however was found in the negative condition between the two groups.”
Results, Dialog Experience and Interview Attitude sub-section, first sentence	“Analysis of the total DEQ score and IAQ score showed that compared to the negative dialogue condition (DEQ: *M* = 32.84, *SD* = 0.99; IAQ: *M* = 20.36, *SD* = 4.2), in the positive condition (DEQ: *M* = 38.33, *SD = *1.24; IAQ: *M* = 30.25, *SD* = 3.21) participants rated their dialogue experience more positively (*F*(1,22) = 252.56, *p*<0.001) and they also had a significantly (*F*(1,22) = 103.6, *p*<0.001) more positive attitude toward the interview.”	“Analysis of the total DEQ score and IAQ score showed that compared to the negative dialogue condition (**DEQ**: *M* = 32.84, *SD* = 1.00; **IAQ**: *M* = 20.38, *SD* = 4.2), in the positive condition (**DEQ**: *M* = 38.32, *SD* = 1.24; **IAQ**: *M* = 30.25, *SD* = 3.21) participants rated their dialogue experience more positively (*F*(1,22) = 252.56, *p* < 0.001) and they also had a significantly (*F*(1,22) = 103.6, *p* < 0.001) more positive attitude toward the interview.”
Results, Dialog Experience and Interview Attitude sub-section, fourth sentence	“As Fig 10 shows, in the negative dialogue condition, the attitude of the higher social anxiety group was significantly lower (*t*(22) = 3.55, *p* = 0.015) than the lower social anxiety group, while in the positive dialogue condition there was no significant difference (*t*(22) = −1.85, *p* = 0.12) between both groups.”	“As Fig 10 shows, in the negative dialogue condition, the attitude of the higher social anxiety group was significantly lower (*t*(5.26) = 3.55, *p* = 0.015) than the lower social anxiety group, while in the positive dialogue condition there was no significant difference (*t*(5.57) = −1.85, *p* = 0.118) between both groups.”

In addition, as a result of the earlier mentioned re-analysis and subsequently identified errors in the statistical analysis or in the reporting, there are errors in Tables 1–4. Please see the correct Tables 1–4 here.

**Table 1:** Comparison between different conditions on SUD score and heart rate.

Measurement		*M1*(*SD*)^a^	*M2*(*SD*)^b^	*t*	*df*	*p*
Condition 1	Condition 2					
**SUD score (0–10)**						
**Neutral**	Blind date	2.38 (0.89)	3.69 (1.01)	-5.55	15	< 0.001
**Neutral**	Job interview	2.38 (0.89)	4.56 (1.03)	-7.89	15	< 0.001
**Blind date**	Job interview	3.69 (1.01)	4.56 (1.03)	-3.42	15	0.011
**Heart rate (bpm)**						
**Neutral**	Blind date	77.2 (11.13)	81.2 (11.30)	-5.7	15	< 0.001
**Neutral**	Job interview	77.2 (11.13)	84.1 (11.03)	-5.72	15	< 0.001
**Blind date**	Job interview	81.2 (11.03)	84.1 (11.03)	-2.7	15	0.048

^a^Mean and standard deviation of condition 1

^b^Mean and standard deviation of condition 2

**Table 2:** Results of univariate analyses with dialogue stressor as within-subjects factor and social anxiety group as between-subjects factor on SUD score, heart rate and audio length.

Factor	Hyp. *df*	Error *df*	*F*	*p*	*η*^*2*^
**SUD score (0–10)**					
**Dialogue stressors**	3.66	80.60	27.66	< 0.001	0.56
**Social anxiety group (high and low)**	1	22	6.84	0.016	0.24
**Dialogue stressors x Social anxiety group**	3.66	80.60	5.68	< 0.001	0.21
**Heart rate**					
**Dialogue stressor**	1.27	27.85	52.75	< 0.001	0.71
**Social anxiety group (high and low)**	1	22	2.61	0.121	0.11
**Dialogue stressors x Social anxiety group**	1.27	27.85	4.14	0.043	0.16
**Audio length**					
**Dialogue stressors**	2.87	63.07	168.07	< 0.001	0.88
**Social anxiety group (high and low)**	1	22	7.24	0.013	0.25
**Dialogue stressors x Social anxiety group**	2.87	63.07	1.30	0.281	0.06

**Table 3:** Comparison between dialog stressor on SUD score rating, heart rate (bpm) and audio length (second).

Measurement		*M1*(*SD*)^e^	*M2*(*SD*)^f^	*t*	*df*	*p*
Condition 1	Condition 2					
**SUD score (0–10)**						
**50% (end)**^a^	0% (C4)^d^	3.88 (0.74)	3.04 (1.27)	-3.75	23	0.006
**0%**	25%	3.04 (1.27)	3.67 (0.92)	3.32	23	0.018
**25%**	50% (avg.)^b^	3.67 (0.92)	3.63 (0.84)	0.28	23	0.999
**50% (avg.)**^b^	75%	3.63 (0.84)	4.42 (1.1)	5.75	23	< 0.001
**75%**	100%	4.42 (1.1)	5.42 (1.1)	-5.54	23	< 0.001
**100%**	50% (end)^c^ (C8)^d^	5.42 (1.1)	4.25 (1.26)	8.14	23	< 0.001
**Heart rate (bpm)**						
**50% (end)**^a^	0% (C4)^d^	82.6 (4.7)	82.3 (3.7)	0.69	23	0.984
**0%**	25%	82.3 (3.7)	84.5 (5.4)	4.16	23	0.002
**25%**	50% (avg.)^b^	84.5 (5.4)	85.8 (7.1)	-2.98	23	0.039
**50% (avg.)**^b^	75%	85.8 (7.1)	90.2 (9.7)	6.83	23	< 0.001
**75%**	100%	90.2 (9.7)	92.6 (10.2)	-8.84	23	< 0.001
**100%**	50% (end)^c^ (C8)^d^	92.6 (10.2)	92.5 (9.9)	0.46	23	0.998
**Audio Length (s)**						
**50% (end)**^a^	0% (C4)^d^	134 (26.0)	177.7 (21.6)	9.7	23	< 0.001
**0%**	25%	177.7 (21.6)	120 (13)	-16.68	23	< 0.001
**25%**	50% (avg.)^b^	120 (13)	101.6 (14.1)	7.06	23	< 0.001
**50% (avg.)**^b^	75%	101.6 (14.1)	71.3 (19)	-10.92	23	< 0.001
**75%**	100%	71.3 (19)	50.6 (13.8)	6.8	23	< 0.001
**100%**	50% (end)c (C8)^d^	50.6 (13.8)	82.7 (16.3)	-10.94	23	< 0.001

^a^Value from the last 50% dialog stressor in the positive condition (C4)

^b^Average value from the first 50% dialog stressor in both negative and positive condition (C1&C5)

^c^Value from the last 50% dialogue stressor in the negative condition (C8)

^d^The control conditions

^e^Mean and standard deviation of condition 1

^f^Mean and standard deviation of condition 2

**Table 4:** Results of univariate analyses with dialogue stressor as within-subjects factor and social anxiety group as between-subjects factor on the individuals’ own valence, arousal and dominance state.

Factor	Hyp. *df*	Error *df*	*F*	*p*	*η*^*2*^
**Valence**					
**Dialogue stressors**	1	22	38.5	< 0.001	0.64
**Social anxiety group (high and low)**	1	22	0.13	0.724	0.01
**Dialogue stressors x Social anxiety group**	1	22	7.07	0.014	0.24
**Arousal**					
**Dialogue stressor**	1	22	19.98	< 0.001	0.48
**Social anxiety group (high and low)**	1	22	4.21	0.052	0.16
**Dialogue stressors x Social anxiety group**	1	22	9.09	0.006	0.29
**Dominance**					
**Dialogue stressors**	1	22	32.2	< 0.001	0.59
**Social anxiety group (high and low)**	1	22	8.75	0.007	0.29
**Dialogue stressors x Social anxiety group**	1	22	4.37	0.048	0.17
