# Supplementary material for: Controlling Social Stress in Virtual Reality Environments
Source: PLoS One. 2014 Mar 26;9(3):e92804. doi: 10.1371/journal.pone.0092804 (PMC3966821; doi:10.1371/journal.pone.0092804)
Supplement: Text S1 — A typical dialogue interaction between a participant (P) and an avatar (A) as observed in the experiment. (DOCX) [file pone.0092804.s001.docx]

**Controlling Social Stress in Virtual Reality Environments**

Dwi HARTANTO, Isabel L. KAMPMANN, Nexhmedin MORINA,

Paul G.M. EMMELKAMP, Mark A. NEERINCX, Willem-Paul BRINKMAN

**Supporting Text S1**

A typical dialogue interaction between a participant (P) and an avatar (A) as observed in the experiment (this dialogue conversation example was performed by a non-native English speaker participant):

**(100%) Positive dialogue style ratio**

**A** question: *“Why do you want to work for our company?”*

**P**: “*Because it matches with my expertise and my passion for sure.*”

**A** response: *“A very good reason indeed! I can see and feel a lot of passion in you now!”*

**A** question: *“What do you know about our company?”*

**P**: “*This company has many brilliant products, from internet service until advanced mobile operating systems, that’s why I would love to work here if I can get hired. Because this what I’m good at!*”

**A** response: *“Well…very good! It seems that you know a lot about this company!”*

**A** question: *“What is your management experience?”*

**P**: “*My previous job was a team leader in a start-up software company, so I have experience in developing high demand software and at the same time maintained my team work that consist of number of software engineers.*”

**A** response: *“You have that experience? That’s a very good experience!”*

**A** question: *“What are you most proud of?”*

**P**: “*Hmmm… [thinking] when I get my master degree with a honour.*”

**A** response: *“That’s a very good achievement indeed! I am really impressed!”*

**A** question: *“What is the biggest mistake that you have ever made?”*

**P**: “*Emm… [thinking] maybe when I didn’t learn a Java language at the first priority. I was a bit sceptics about java at that time and more focused on WPF instead. Then Android came and turned around the table, where java is a must. Well, now I’m a big fan of Android operating system. So, the only thing to catch is I need to learn java, bit by bit. But finally I can catch the learning process in the end. However, I’ve just regretted and imagine if I’m not that late to learn java. But you know, now I’m not that bad after all [chuckle], but yeah just regret it. A bit.*”

**A** response: *“Interesting! So you have learned a lot starting from that moment then.”*

**A** question: “*How do you handle stress?*”

**P**: “*Em… [thinking]* *Well, sure! I have a good self-management and well organize person. I keep track on every task and trying to deliver it as soon as possible. So, with this approach I can prevent myself from deadline pressure and stress over my work. And I’m glad it still works until now!*”

**A** response: “*Hmmm… [amazed tone] A very good approach indeed! I’m so impressed!*”

**A** question: “*Tell me about your hobbies please?*”

**P**: “*Well, I like photography. So, normally during the weekend or holiday, I go out and try to start capturing a nice object or rare moments wherever I go. And this hobby sometimes give you money [chuckle], of course by selling it to the newspaper agency. Or you can share it to the world and let other people enjoy the beauty that you’d just captured. And when a lot of people like it, that’s another priceless moment that you can enjoy.*”

**A** response: “*A very interesting hobby indeed! A really nice one!*”

**(100%) Negative dialogue style ratio**

**A** question: *“Are you sure you want to work for our company?”*

**P**: “*Yes, I like this company.*”

**A** response: *“That’s all? Nothing else? It seems you’re not so serious about working here!”*

**A** question: *“Are you sure you know something about this company?”*

**P**: “*Hmmm yes, more less. Your search engine is world famous.*”

**A** response: *“Hmm… [doubting tone] You should have at least visited our website before coming here!”*

**A** question: *“I don’t think that you have management experience, do you? Or am I wrong?”*

**P**: “*Mmm… [thinking]* *I have for sure, when I was in internship, I have a role to take on some of software development modules. My supervisor promoted me to be the organizer, so one of my task was organizing the software release together with couple of my colleagues. I manage to deliver the package modules on time and keep my team in a good shape.*”

**A** response: *“Oh, well, it seems that you have nothing there then!”*

**A** question: “*I’m sure that you have nothing to show, but in case I’m wrong, can you tell me what you are most proud of?”*

**P**: “*Mmm… [thinking] Maybe when I won a national software competition while I’m still in my bachelor.*”

**A** response: *“That’s all? Doesn’t look so good to me. But never mind, let’s continue!”*

**A** question: *“I assume that you have made a lot of mistakes in the past, can you tell me the biggest one?”*

**P**: “*Well, Mmmm… [thinking]* *Maybe when I lost my project documents when there’s an option to make a backup regularly. I was thinking that I don’t have so much time to do a backup at that time, so I just keep ignoring the pop up message. Until there’s a hardware failure and wipe away my documents.*”

**A** response: *“That’s a stupid one! Around here we don’t tolerate this kind of mistakes!”*

**A** question: “*Hmmm… [doubting tone] Can you handle stress? And how?”*

**P**: “*Mmm… [thinking]* *I think simply by avoiding it. You need to have a good time management to do this.*”

**A** response: *“Hmm… [doubting tone] That’s all? That’s doesn’t look so good to me!”*

**A** question: “*Do you have any hobbies?”*

**P**: “*Yes. I like doing sport exercise, especially jogging or running. And every weekend I spend a couple of hours jogging just to keep myself always in a good shape.*”

**A** response: “*Hmm… Typical! [long breath taking] yeah, most of people will answer that!*”
